# Copy Number Variation on *ABCC2-DNMBP* Loci Affects the Diversity and Composition of the Fecal Microbiota in Pigs

**DOI:** 10.1128/spectrum.05271-22

**Published:** 2023-05-31

**Authors:** Yuliaxis Ramayo-Caldas, Daniel Crespo-Piazuelo, Jordi Morata, Olga González-Rodríguez, Cristina Sebastià, Anna Castello, Antoni Dalmau, Sebastian Ramos-Onsins, Konstantinos G. Alexiou, Josep M. Folch, Raquel Quintanilla, Maria Ballester

**Affiliations:** a Animal Breeding and Genetics Program, Institute of Agrifood Research and Technology, Caldes de Montbui, Spain; b Centro Nacional de Análisis Genómico, Centre for Genomic Regulation, Barcelona Institute of Science and Technology, Barcelona, Spain; c Plant and Animal Genomics Program, Centre for Research in Agricultural Genomics, Consejo Superior de Investigaciones Científicas (CSIC)-Institute of Agrifood Research and Technology-Autonomous University of Barcelona-UB, Bellaterra, Spain; d Animal and Food Science Department, Autonomous University of Barcelona, Bellaterra, Spain; e Animal Welfare Program, Institute of Agrifood Research and Technology, Girona, Spain; University of Valencia

**Keywords:** diversity, microbiota, modulators, porcine, structural variants

## Abstract

Genetic variation in the pig genome partially modulates the composition of porcine gut microbial communities. Previous studies have been focused on the association between single nucleotide polymorphisms (SNPs) and the gut microbiota, but little is known about the relationship between structural variants and fecal microbial traits. The main goal of this study was to explore the association between porcine genome copy number variants (CNVs) and the diversity and composition of pig fecal microbiota. For this purpose, we used whole-genome sequencing data to undertake a comprehensive identification of CNVs followed by a genome-wide association analysis between the estimated CNV status and the fecal bacterial diversity in a commercial Duroc pig population. A CNV predicted as gain (DUP) partially harboring ABCC2-DNMBP loci was associated with richness (*P* = 5.41 × 10^−5^, false discovery rate [FDR] = 0.022) and Shannon α-diversity (*P* = 1.42 × 10^−4^, FDR = 0.057). The *in silico* predicted gain of copies was validated by real-time quantitative PCR (qPCR), and its segregation, and positive association with the richness and Shannon α-diversity of the porcine fecal bacterial ecosystem was confirmed in an unrelated F1 (Duroc × Iberian) cross. Our results advise the relevance of considering the role of host-genome structural variants as potential modulators of microbial ecosystems and suggest the ABCC2-DNMBP CNV as a host-genetic factor for the modulation of the diversity and composition of the fecal microbiota in pigs.

**IMPORTANCE** A better understanding of the environmental and host factors modulating gut microbiomes is a topic of greatest interest. Recent evidence suggests that genetic variation in the pig genome partially controls the composition of porcine gut microbiota. However, since previous studies have been focused on the association between single nucleotide polymorphisms and the fecal microbiota, little is known about the relationship between other sources of genetic variation, like the structural variants and microbial traits. Here, we identified, experimentally validated, and replicated in an independent population a positive link between the gain of copies of *ABCC2-DNMBP* loci and the diversity and composition of pig fecal microbiota. Our results advise the relevance of considering the role of host-genome structural variants as putative modulators of microbial ecosystems and open the possibility of implementing novel holobiont-based management strategies in breeding programs for the simultaneous improvement of microbial traits and host performance.

## INTRODUCTION

Gut microbiomes have a profound impact on many aspects of pig health, such as the modulation of metabolic functions, physiological processes, and relevant porcine traits like growth (1), feed efficiency (2, 3), and immunocompetence (4). Pig gut microbiota composition is highly variable among individuals. Host-microbiome interactions are mediated by both environmental and host factors. Among them, genetic variation in the pig genome can modulate, in a taxa-specific manner, the composition and function of the pig gut eukaryotic and prokaryotic communities. Several studies have reported low to medium heritability values for pig gut microbiota composition (2, 5, 6) that varies according to specific taxon or taxonomic levels. In addition, quantitative trait loci (QTLs), genetic variants, and candidate genes linked to microbial traits have been identified in pigs (7–10). However, since previous studies were focused on the association between single nucleotide polymorphisms (SNPs) and microbial traits, little is known about the relationship between the fecal microbiota and structural variants in the porcine genome.

Copy number variants (CNVs) are structural variants that produce a change in the number of copies (gain or loss) of a genomic region. Compared to SNPs, CNVs involve large DNA segments that span a significant proportion of the genome and account for greater genomic variability than SNPs. Consequently, CNVs are a relevant source of genetic variation that contribute to evolutionary adaptations and variation in gene expression and phenotypic traits in human and domestic animals (11, 12). In humans, gain of copies of the salivary amylase (*AMY1*) gene was associated with oral and gut microbiome composition (13). In this seminal study, Poole et al. found that individuals with greater number of copies of *AMY1* showed greater levels of salivary Porphyromonas, followed by an increased abundance of resistant starch-degrading microbes in the gut (13). We hypothesized that as in humans, CNVs are likely to contribute to gut microbial variability in animals.

In pigs, CNVs have been extensively characterized (12, 14–16) and have been found to be associated with a variety of traits such as coat color (17, 18), fatty acid composition (19), and growth and reproductive traits (20–22). However, to the best of our knowledge, associations between CNVs and microbial traits have not been documented in livestock. Consequently, the putative modulatory role of CNVs in the diversity, composition, and function of livestock gastrointestinal microbiota remains to be elucidated. The main goal of this study was to assess the effect of porcine CNVs on the diversity and composition of pig fecal microbiota.

## RESULTS

### Detection of copy number variants and analysis.

**FIG 1:**
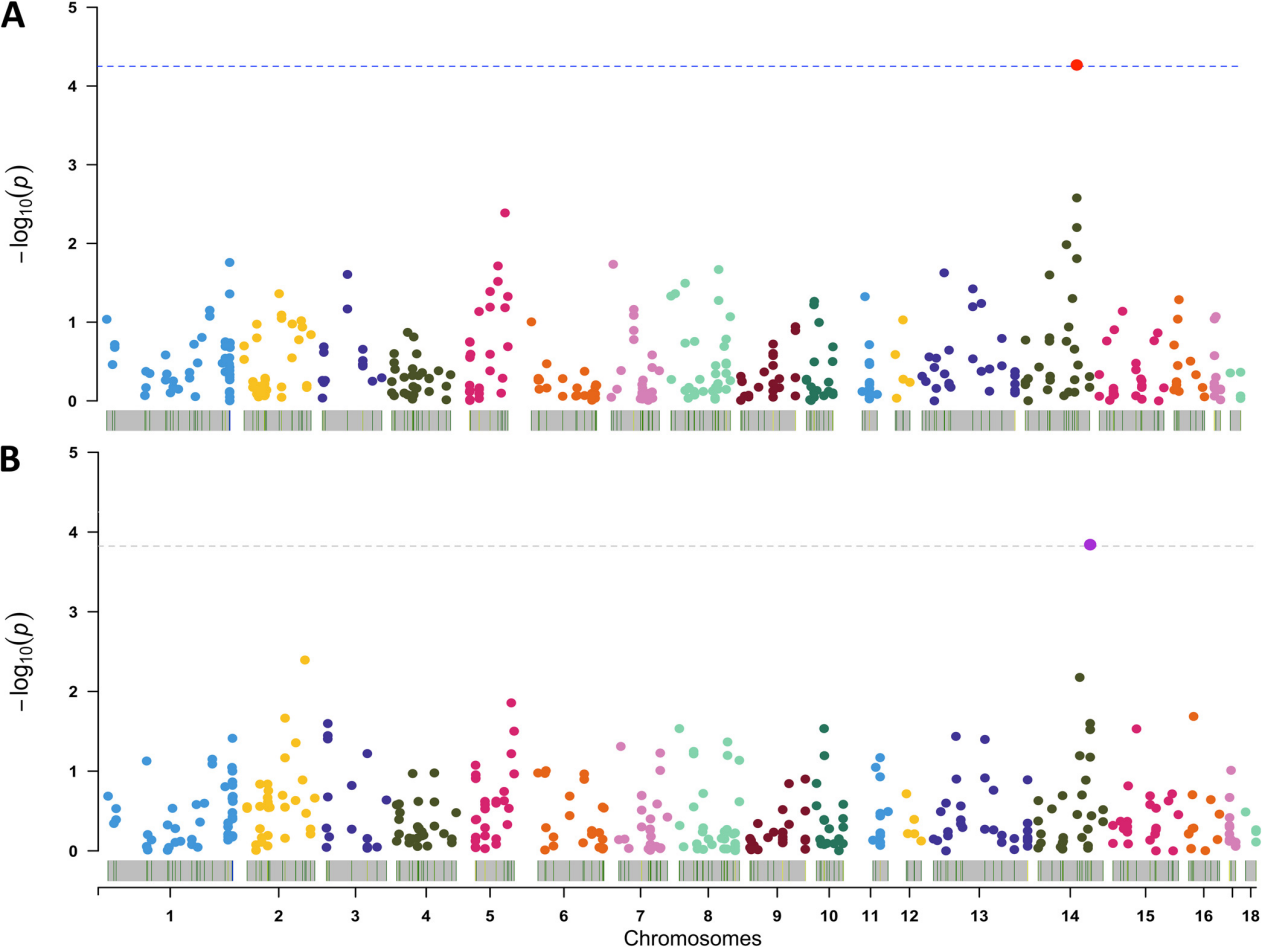
Results from the association analyses of copy number variants (CNVs) identified across the pig genome with gut bacterial richness (A) and Shannon α-diversity (B). The *x* axis represents the CNV position in the pig autosomal chromosomes (1 to 18), and the *y* axis reflects the significance level represented as the –log_10_ (*P*). The dashed lines correspond to the significance threshold after multiple test correction. The red and purple points on SSC14, Sus scrofa chromosome 14 represent the CNV comprising ABCC2-DNMBP genes.

In this study, we used whole-genome sequencing data from 100 healthy 60-day-old Duroc pigs to undertake a comprehensive identification of CNVs. A total of 1,292 CNVs distributed across 531 copy number variant regions (CNVRs) on autosomal pig chromosomes were identified (Table S1). After quality control, 1,005 CNVs grouped into 291 CNVR, presented in at least the 5% of the samples, were used for the association analysis. Among them, a CNV predicted as gain located on CNVR454 (SSC14:111000000-111075999) that partially contain the ATP-binding cassette subfamily C member 2 (*ABCC2*) and the dynamin-binding protein (*DNMBP*) genes showed a significant association with richness (*P* = 5.41 × 10^−5^) and Shannon α-diversity (*P* = 1.42 × 10^−4^) (Fig. 1).

### Validation by quantitative PCR.

The *in silico* identification of CNVs may result in both false-positive and -negative results (14, 23). To confirm the presence of the CNVR454 in our animal material, we conducted a quantitative PCR (qPCR) assay with primers located on the *ABCC2* gene. The *in silico* predicted genotype was confirmed by qPCR in 46 of 48 Duroc samples, corresponding to a precision of 95.83%. To be noted, all the 24 samples *in silico* predicted as DUP presented the gain in number of copies. Thus, deviations from the diploid status were observed in 2 of 24 animals, in which variation in the number of copies was not predicted by Control-FREEC (24).

**FIG 2:**
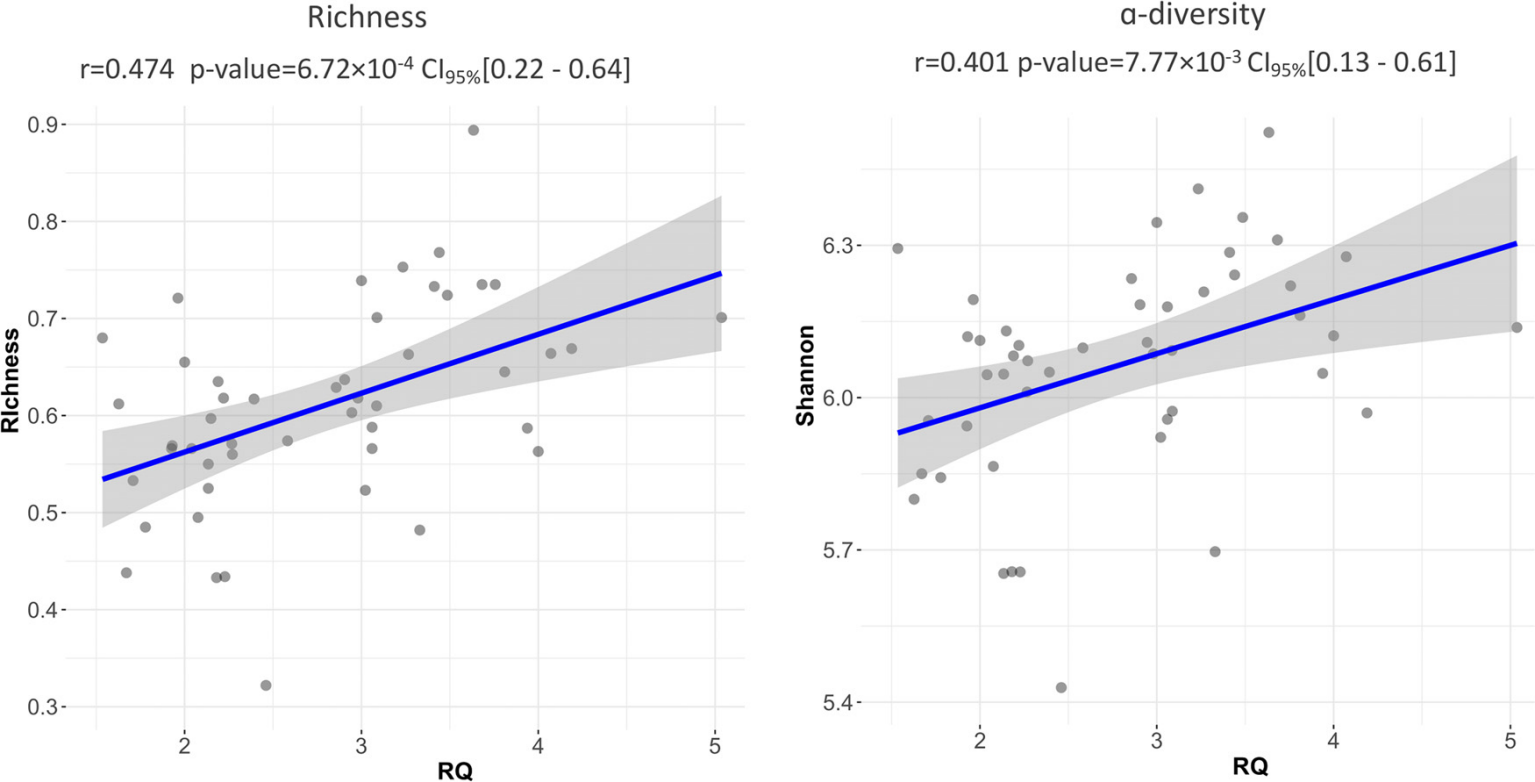
Relationship between the CNV relative quantification (RQ) of the number of copies with the richness and Shannon α-diversity index.

Since the presence of false-negative samples for CNV status could affect the genome-wide association studies (GWAS) results, we evaluated the correlation between the CNV relative quantification by qPCR (2N = 22 versus DUP = 26) and the diversity index. In agreement with the CNV-GWAS, qPCR results corroborated that DUP pigs had significantly greater richness (*P* = 1.8 × 10^−3^) and α-diversity values (*P* = 3.8 × 10^−3^) (Table 1). Furthermore, relative quantification (RQ) of the number of copies was positively correlated with the richness (*r* = 0.474, *P* = 6.72 × 10^−4^) and the α-diversity (*r* = 0.401, *P* = 7.77 × 10^−3^) (Fig. 2). We also observed that compared to their diploid counterparts, DUP samples had lower β-diversity (2N = 0.419 versus DUP = 0.375, *P* = 6.36 × 10^−3^), suggesting a more homogeneous fecal microbial ecosystem (i.e., more similar microbiota between samples). Furthermore, a positive relationship between the RQ of the number of copies and the nucleotide variability of the CNV genomic interval estimators ATajima (*r* = 0.428, *P* = 2.4 × 10^−3^) and RTajima (*r* = 0.71, *P* = 1.26 × 10^−8^). The high correlation observed between RTajima and the RQ values (Fig. S1) can be explained by the characteristic of RTajima statistic, which gives more importance to intermediate frequencies observed in the whole population. Finally, a positive and significant correlation was observed with RTajima between the richness (*r* = 0.27, *P* = 0.01) and the Shannon α-diversity (*r* = 0.38, *P* = 6.9 × 10^−3^) (Fig. S1).

**TABLE 1:** Description of the diversity index of the Duroc and F1 (Duroc × Iberian) cross

Population	Data set (*n*)	Groups	Richness (SD)	α-Diversity (SD)
Duroc (discovery)	Complete (100)		604.92 (98.17)	6.05 (0.22)
	Validate qPCR (48)	Diploid	553.73^*a*^ (92.71)	5.95^*a*^ (0.21)
		DUP	657.85^*b*^ (89.70)	6.16^*b*^ (0.17)
Duroc × Iberic (validation)	Complete (285)		459.65 (104.33)	5.82 (0.25)
	Validate qPCR (24)	Diploid	292.36^*c*^ (67.18)	5.36^*c*^ (0.27)
		DUP	543.19^*d*^ (158)	6.06^*d*^ (0.33)

a,b,c,d Within a column of samples validated by quantitative PCR (qPCR), values without a common superscript differ (*P* < 0.05).

**FIG 3:**
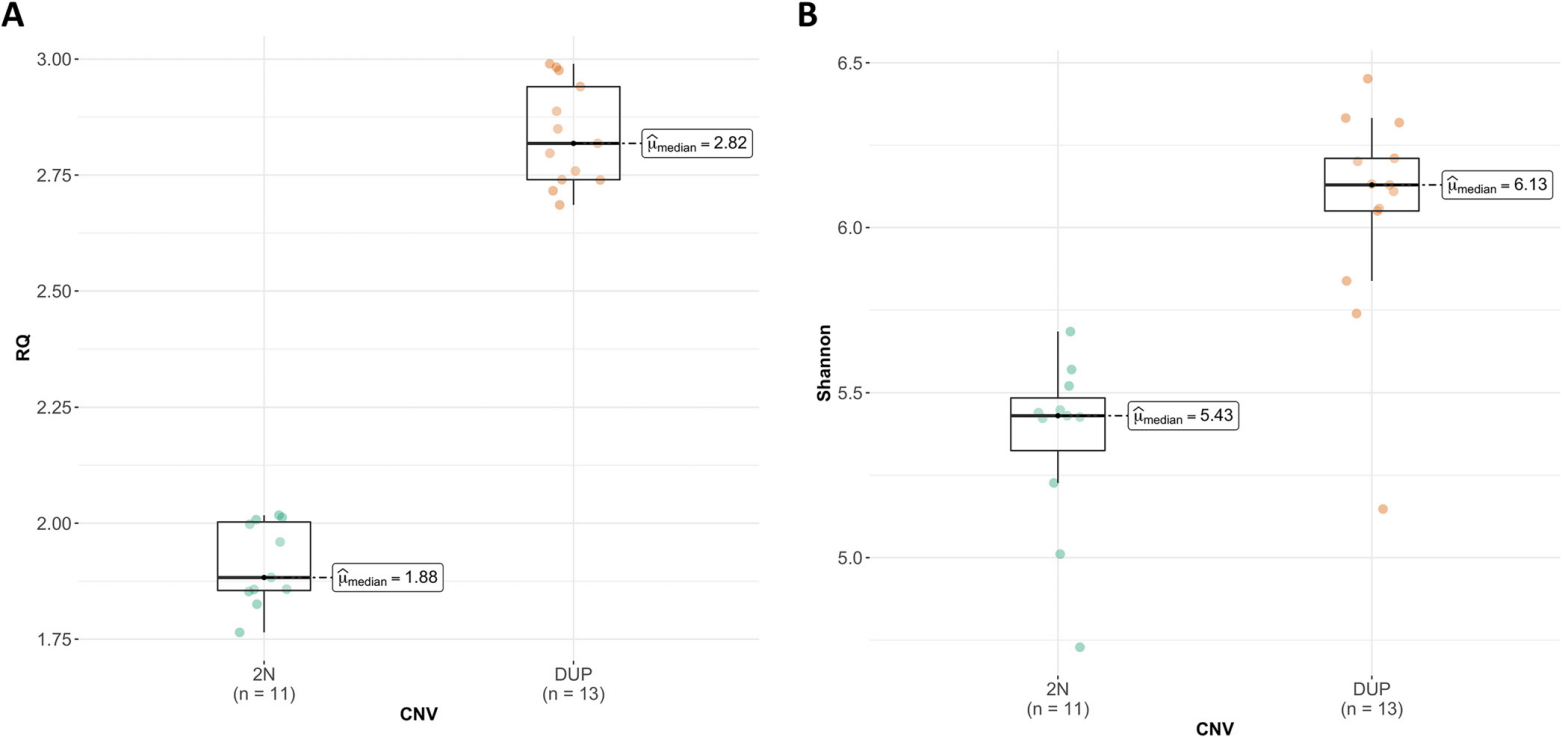
Results from the replication analysis comparing (A) mean RQ and (B) Shannon α-diversity of *ABCC2-DNMBP* loci in the F1 Duroc × Iberian cross. Green dots represents diploid (2N) samples, and orange dots represent DUP samples.

Remarkably, variation in the number of copies of the *ABCC2-DNMBP* loci was also segregating in an unrelated commercial F1 Duroc × Iberian crossbred pigs, with 13 of 24 pigs showing a gain of copies. Nonsignificant differences between males and females were observed (*P* > 0.05). The 12 samples with the highest diversity index (Table 1) presented the gain in the number of copies of the *ABCC2-DNMBP* loci. Meanwhile, the diploid status was observed in 11 of the 12 samples with the lowest diversity. Moreover, despite differences on genetic background, age, or other environmental factors such as diet of farm of origin, the association between the *ABCC2-DNMBP* loci and the fecal microbial diversity was replicated in the F1 Duroc × Iberian cross (Table 1; Fig. 3). Indeed, in both Duroc and F1 Duroc × Iberian cross data sets, the qPCR reaffirmed that a gain of copies of the *ABCC2-DNMBP* loci was positively associated with the richness and Shannon α-diversity of the pig fecal microbiota.

### Microbial signatures linked to variation in the number of copies.

**FIG 4:**
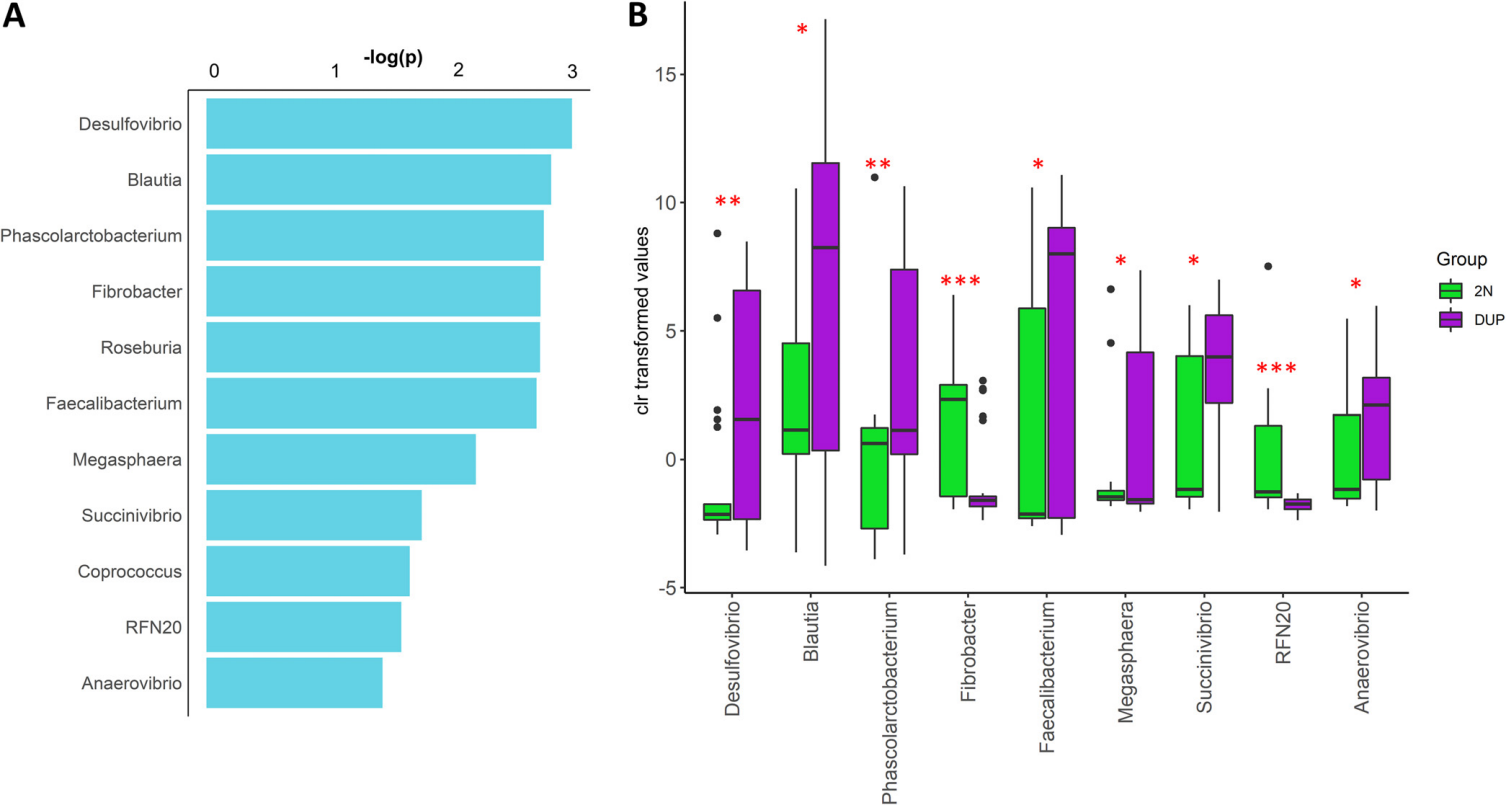
Results from microbial signature analyses at genus level. (A) Taxa-set enrichment. (B) Patterns of differential abundance analysis between DUP (*N* = 26) and 2N (*N* = 22) pigs in the purebred Duroc population. The mean values for the two groups are significantly different. *, *P* < 0.05; **, *P* < 0.01; **, *P* < 0.001.

**FIG 5:**
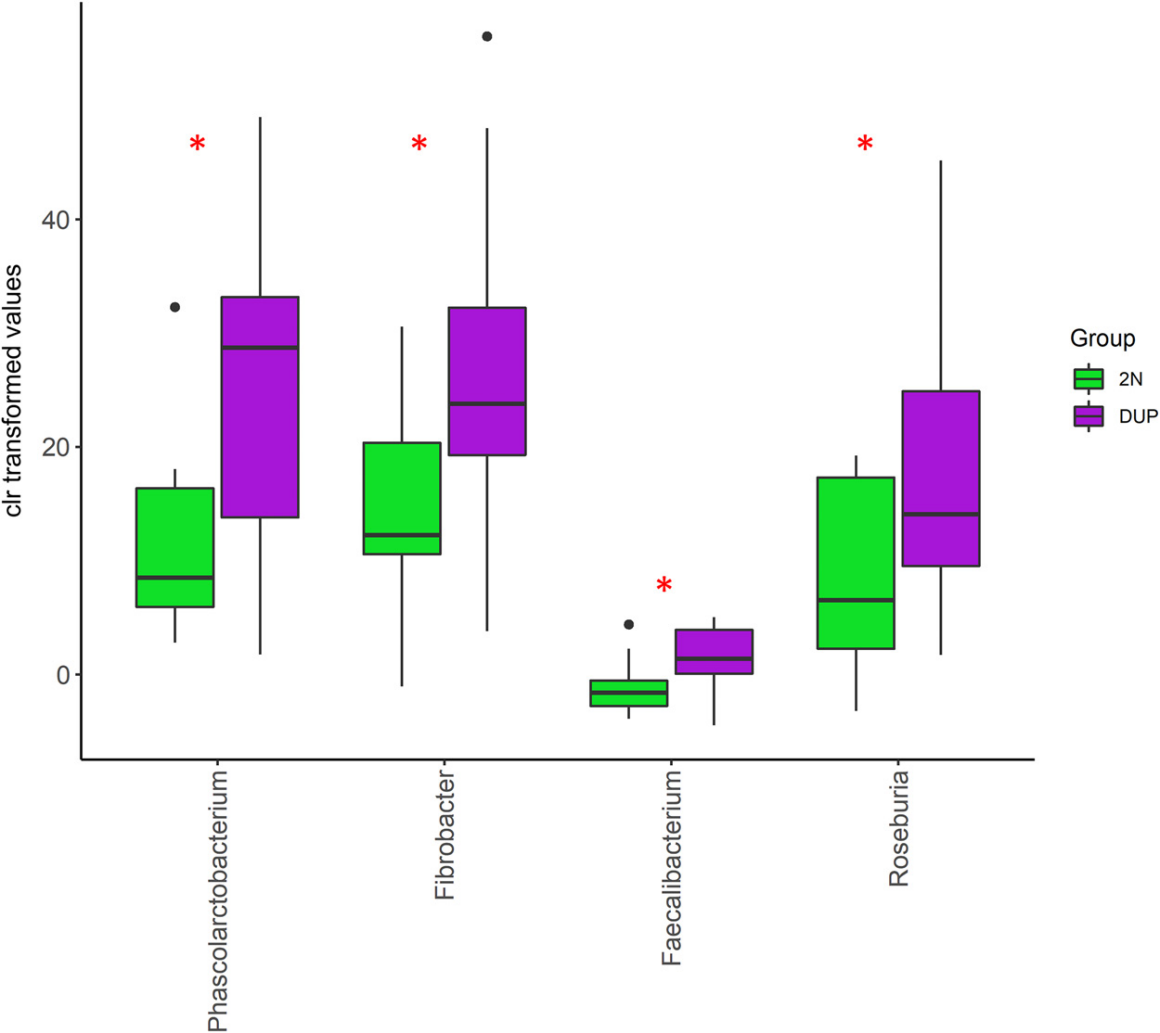
Differential abundance patterns at genus level between DUP (*N* = 13) and 2N (*N* = 11) samples in the F1 Duroc × Iberian crossbred population. The mean values for the two groups are significantly different. *, *P* < 0.05.

The results from the supervised classification model showed that the relative abundance of 122 of 3,052 ASVs allowed the classification (mean accuracy of 0.67) between groups of samples (Table S2). The taxa-set enrichment analysis pointed out a higher overall discriminant importance of ASVs members of the Desulfovibrio, Blautia, Phascolarctobacterium, Fibrobacter, Roseburia, Faecalibacterium, Megasphaera, Succinivibrio, Coprococcus, RFN20, and Anaerovibrio genera (Fig. 4A). Furthermore, supporting their discriminative role, we observed that compared to their diploid counterparts, the fecal microbiota of DUP pigs exhibited a higher relative abundance (false discovery rate [FDR] < 0.05) of the Desulfovibrio, Blautia, Phascolarctobacterium, Faecalibacterium, Megasphaera, Succinivibrio, and Anaerovibrio genera but lower relative abundance of the Fibrobacter and RFN20 genera (Fig. 4B). To be noted, the results obtained from the differential abundance analysis done on the unrelated F1 Duroc × Iberian crossbred population confirmed a higher relative abundance of the Phascolarctobacterium, Roseburia, and Faecalibacterium genera in the fecal microbiota of samples with a gain of copies of the *ABCC2-DNMBP* loci (Fig. 5).

## DISCUSSION

In this study, we report, for the first time in a livestock species, a CNV partially containing the *ABCC2* and *DNMBP* genes associated with the diversity and composition of the pig fecal microbiota. *ABCC2* encodes a multidrug resistance-associated protein 2 (MRP2) that plays a relevant role in preserving hepatic and intestinal homeostasis (25). ABCC2 is involved in the excretion of conjugated bile acids (BAs), bilirubin, and xenobiotics and the transport of other organic anions (26, 27). In pigs, *ABCC2* has been reported as coassociated with the intramuscular profile of fatty acid composition in an Iberian × Landrace cross (28). In addition, the genomic interval harboring the *ABCC2-DNMBPloci* overlapped with QTLs associated with the muscle profile of palmitic (QTLId: 95385), stearic (QTLId: 95386), and palmitoleic (QTLId: 95387) fatty acids content in a Duroc × (Landrace × Yorkshire) cross (29). In other species such as mice, rats, or humans, mutations in *ABCC2* are related to hereditary liver diseases. *Mrp2*^−/−^mice are viable (30, 31) but, like *ABCC2*-knockout rats, showed chronic hyperbilirubinemia followed by a reduction in biliary excretion of bilirubin glucuronides (30, 32). Meanwhile, mutations in the human *ABCC2* gene result in Dubin-Johnson syndrome, an autosomal recessive disorder characterized by a defect in the transport of endogenous and exogenous anionic conjugates from hepatocytes into the bile (33). It is noteworthy that a genomic duplication of 5,299 bp comprising exons 24 and 25 of human *ABCC2* gene was predicted to result in the insertion of a premature stop codon (34).

Based on the known role of *ABCC2* in the excretion of bilirubin and conjugated BAs, we propose that variations in the number of copies of *ABCC2* may influence gut levels of these substances in the gut. Previous research has also demonstrated bidirectional cross talk between the gut microbiota and the metabolism of bilirubin and conjugated BAs, which may be further influenced by variations in the number of copies in*ABCC2*. For example, bilirubin can regulate the composition of gut microbiota by being potentially toxic toward Gram-positive bacteria, while promoting the proliferation of Gram-negative species (35). In a similar way, a higher BA tolerance is evidenced by Gram-negative bacteria (36). In agreement with these studies, the fecal microbiota of DUP samples showed a higher relative abundance of Gram-negative bacteria, such as members of the Desulfovibrio, Phascolarctobacterium, Faecalibacterium, Succinivibrio, and Anaerovibrio genera. On another note, gut microbiota composition can regulate BA and bilirubin production and signaling. In addition, conjugated BAs can have a protective role on gut barrier integrity (37). The oral administration of two major conjugated BAs, tauro-cholic acid and β-tauro-murocholic acid, increased the richness of neonatal small intestinal microbiota with a positive effect on the postnatal microbiota maturation (38). It is noteworthy that among the top discriminant ASVs, we observed butyrate-producer species with a potentially beneficial effect on the host, such as Blautia obeum (*ASV2433*, *ASV2171*, *ASV2278*), Faecalibacterium prausnitzii (*ASV2371*, *ASV2378*, *ASV2396*), and Roseburia sp001940165 (*ASV1822*). Interestingly, the genome of all these species encodes bile salt hydrolases (BSHs, EC 3.5.1.24) (39, 40), enzymes that mediate the primary BA deconjugation and successive conversion to secondary BAs, thereby partly determining the amount of secondary BAs in the colonic epithelium, which in turn act as signaling molecules mediating different metabolic processes interconnected with health and diseases (41, 42).

The CNVR454 also included *DNMBP*, a gene that regulates the structure of apical junctions through F-actin organization in epithelial cells (43). *DNMBP* is also involved in luminal morphogenesis and enterocyte polarization (44, 45), thus potentially contributing to the function and homeostasis of the intestinal epithelial barrier (IEB). In fact, the cross talk between IEB and the gut microbiota is crucial for the maintenance of intestinal homeostasis. For example, enterocytes, which are the most abundant population among intestinal epithelial cells, express a range of pattern recognition receptors for sensing the microbe-associated molecular patterns. Further, enterocyte apex is covered by thousands of microvilli that are vital in colonic wound repair and the transport of molecules and nutrients such as bile salts, electrolytes, and vitamins (46–49). Interestingly, compared with conventional piglets, germfree (GM) pigs displayed aberrant intestinal morphology with longer villi and shorter crypts. Meanwhile, the oral administration of commensal bacteria increased crypt depth and induced enterocyte brush border microvilli enzyme activities on these GM piglets (50–53). Therefore, considering the functional roles of *DNMBP* in the IEB, we cannot rule out the contribution of *DNMBP* to the modulation of the diversity and composition of the pig gut microbiota.

Altogether, our results pinpointed a positive association of the variation in the number of copies of the *ABCC2-DNMBP* loci with the richness, α-diversity, and composition of the pig fecal microbial ecosystems. Such findings open the possibility of modulating the fecal microbiota, which has emerged as a promising breeding or therapeutic tool to optimize livestock production efficiency, animal health, and well-being. Greater gut microbial diversity is usually desired and generally accepted as an indicator of a resilient microbial ecosystem, gut, and host health. Indeed, a diverse and healthy gut has a positive effect on the absorption of dietary nutrients, feed efficiency, and animal well-being.

We are aware of some limitations of our study like the limited taxonomic resolution achieved by targeting the V3-V4 16S rRNA genomic region with short-read sequencing. We are also aware of the convenience of performing further analyses to confirm the raised hypotheses by assessing the metabolic profile of BAs, as well as evaluating the role of the CNV on gene expression (at both the microbial and host levels) of genes involved in BA metabolism. Despite these limitations, our findings contribute to the understanding of host-microbiome interactions. Moreover, our results open the possibility of breeding the holobiont via the incorporation of this source of variation on custom-made arrays that can be used in routine genotyping tasks applied to porcine breeding programs and, together with nutritional or management strategies, will favor the simultaneous improvement of microbial traits, gut health, and host performance.

### Conclusions.

Here, we report the first study exploring associations between porcine CNV and the diversity and composition of the pig fecal microbiota. In an unrelated population, we identified, functionally validated, and replicated a positive association between the gain of copies of *ABCC2-DNMBP* loci and the composition and diversity of the pig fecal microbiota. These results suggest a role for the host-genome structural variants in the modulation of microbial ecosystems and open the possibility of including CNVs in selection programs to simultaneously improve microbial traits, gut health, and host performance.

### Ethics approval and consent to participate.

The animal care and experimental procedures were carried out following the institutional guidelines for good experimental practices and the Spanish Policy for Animal Protection RD 53/2013, which meets the European Union Directive 2010/63/EU for protection of animals used in experimentation, and were approved by the Institute of Agrifood Research and Technology Ethical Committee. Consent to participate is not applicable in this study.

## MATERIALS AND METHODS

### Animal samples.

The samples employed in this study are a subset of pigs reported in references 9 and 54. In brief, a total of 100 healthy piglets (50 males and 50 females) aged 60 ± 8 days from a commercial Duroc pig line were used as a discovery data set (Table 1). All animals were raised on the same farm and had *ad libitum* access to the same commercial cereal-based diet. Furthermore, a subset of 24 unrelated F1 (Duroc × Iberian) crossbred pigs (300 days old, 12 males and 12 females) with phenotypically extreme fecal microbial diversity index (12 high and 12 low) from reference 7 were employed as an independent validation data set (Table 1).

### Microbial DNA extraction, sequencing, and bioinformatics analysis.

Fecal samples were collected from the Duroc piglets at 60 ± 8 days of age, and microbial DNA was extracted with the DNeasy PowerSoil kit (Qiagen, Hilden, Germany) following the manufacturer’s recommendations. Extracted DNA was sent to the University of Illinois Keck Center for paired-end (2 × 250 bp) sequencing on an Illumina NovaSeq (Illumina, San Diego, CA, USA). The 16S rRNA gene fragment was amplified using the primers V3_F357_N (5′-CCTACGGGNGGCWGCAG-3′) and V4_R805 (5′-GACTACHVGGGTATCTAATCC-3′). The sequences were analyzed with QIIME2 (55); barcode sequences, primers, and low-quality reads (Phred score < 30) were removed. The quality control process also trimmed sequences based on expected amplicon length and removed chimeras. Afterwards, the sequences were clustered into amplicon sequence variants (ASVs) at 99% identity. ASVs were classified to the lowest possible taxonomic level based on a primer-specific trained version of GreenGenes2 Database (released October 2022) (56, 57). Before the estimation of the diversity indices, to correct for the sequencing depth, the samples were rarefied at 10,000 reads. The diversity metrics at ASV levels were estimated with the vegan R package version 2.6-2 (58). The α-diversity was evaluated with the Shannon index (59), and the β-diversity was assessed using the Whittaker index (60). The graphical representations of means comparison were done with the *ggstatsplot* R library (61).

### Host-genome data analysis and CNV calling.

Simultaneously with fecal sampling, blood was collected at 60 ± 8 days of age via the external jugular vein. Host genomic DNA was extracted from blood using the NucleoSpin blood protocol (Macherey-Nagel). The whole genome was paired-end sequenced (2 × 150 bp) in an Illumina NovaSeq 6000 platform (Illumina) at the Centro Nacional de Análisis Genómico (Centre for Genomic Regulation, Barcelona, Spain). Reads were mapped to the porcine reference assembly Sscrofa.11.1 with BWA-MEM 0.7.17 (62). Alignment files containing only properly paired, uniquely mapping reads without duplicates were processed using Picard (63) to add read groups and to remove duplicates. Variant calling was performed with the Haplotype Caller tool from the Genome Analysis Toolkit (GATK 4.1.8.0) (64). Applying GATK best practices, variants with minimum read depth of 5 on at least one sample were retained. Joint genotyping was conducted with combined genomic variant call format (gVCFs). Functional annotations were added using SnpEff v.5 (65) against the Sscrofa.11.1 reference database. CNV prediction was performed with Control-FREEC 11.5 (24), using a pool of samples as CNV baseline and using intervals of 20 kb. CNV calls from all samples that were less than 10 kb apart were merged with Survivor (66). Individual CNV calls were combined into copy number variant regions (CNVRs) following the reciprocal overlap approach (11) with CNVRange (67). Therefore, contiguous CNVs intervals with at least 50% of mutual overlap were merged into the same CNVR.

Nucleotide diversity pattern estimates per individual were calculated considering the SNPs present in each individual separately. Here, we tested two estimators: (i) Tajima’s theta estimator (π or nucleotide diversity, called ATajima here), that is simply the number of variants present in the individual; and (ii) RTajima estimator, which considers the frequency observed in the entire sample but was calculated given the SNPs present in each individual. This estimate is calculated with the same principles that are in ATajima but need to be corrected by the probability that only a portion of the total SNPs from a sample is present in each of the samples (by using a hypergeometrical distribution). That is, for each individual, this is an estimate of the nucleotide diversity of the entire population if the individuals behave as if they belonged in a stationary neutral population. Specifically, for this new variability estimate expression, the calculation for a single diploid individual is as follows:

$$\hat{\theta }=\frac{1}{\sum_{i=1}^{n-1}(n-i)}\overset{n-1}{\underset{i=1}{\sum }}(n-i)i{\text{ξ}}_{i}{\left(1-\frac{\left(\begin{array}{c} n-i\\ 2 \end{array}\right)}{\left(\begin{array}{c} n\\ 2 \end{array}\right)}\right)}^{-1}$$

θ is the estimate of population variability using a single individual, *n* is the number of samples in the population (2× number of individuals), and ξ*_i_* is the number of SNPs observed in this individual that are at frequency *i* in the whole population. Note that this expression is constructed using an approach (68) to estimate nucleotide diversity (left-side expression after equality) and the hypergeometric correction (right-side expression).

The data set in VCF format was converted to FASTA and from FASTA to transposed FASTA (tFASTA). This tFASTA file was read with the software mstatspop (https://github.com/CRAGENOMICA/mstatspop) to obtain the frequencies of SNPs from each of the pigs at the desired region. Finally, we calculated RTajima per fragment using self-made R scripts. All these estimates were finally divided by the effective length size of the studied region to obtain comparative estimates per nucleotide.

### CNV-wide association analysis.

A genome-wide association analysis between the estimated CNV status and fecal bacterial diversity index was done with GCTA (69) using the following mixed model:

$$y_{\mathrm{ijk}}={\text{sex}}_{j}\;+\;b_{k}+u_{i}\;+\;{\text{cnv}}_{\mathrm{li}}\;+\;e_{\mathrm{ijk}}$$

where *y_ijk_* corresponds to the microbial index under scrutiny (richness or Shannon α-diversity) of the *i*th individual animal of sex *j* in the *k*th batch; sex*_j_* and *b_ki_* correspond to the systematic effects of *j*th sex (two levels) and *k*th batch (three levels), respectively; *u*_i_ is the random additive genetic effect of the *i*th individual, collectively distributed as *u* ~ *N* (0, *G* ${\text{σ}}_{u}^{2}$) where ${\text{σ}}_{u}^{2}$ is the additive genetic variance, and *G* is the numerator of the genomic relationship matrix calculated using the autosomal SNPs; cnv*_li_* is the genotype (recoded as 11 = loss, 12 = diploid, and 22 = gain) for the *l*th CNV of the *i*th individual, and *e_ijk_* is the residual. To correct for multiple testing, the FDR was calculated with the *p.adjust* function of R.

### Quantitative real-time PCR.

Real-time qPCR was used to validate the CNV on the *ABCC2* gene in a total of 72 samples, including a subset of 48 Duroc samples (24 diploid and 24 *in silico* predicted as DUP), and 24 unrelated F1 Duroc × Iberian cross (Table 1). The CNV breakpoint was re-estimated with Manta version 1.6.0 (70). All primers were designed using the Primer Express 2.0 software (Applied Biosystems). The pair of primers ABCC2_CNV_F (5′-TGGCATCATTTATGTGGCTGTT-3′) and ABCC2_CNV_R (5′-AGGAAGGAGCTTGGGCTTTTA-3′) amplify a specific region of the *ABCC2* gene (exon 25-intron 25 of transcript ABCC2-201 with Ensembl ID ENSSSCT00000011534.5) containing the CNV, while the pair of primers ABCC2_F (5′-TGGACAAGAACCAGAGTCAAAGC-3′) and ABCC2_R (5′-ACATAGAGCGCATTTGAACGAA-3′) amplify a region outside the estimated CNV (exon 7-intron 7 of transcript ABCC2-201 with Ensembl ID ENSSSCT00000011534.5) breakpoint that was used as single copy control region. The 2 − ΔΔ*Ct* method for RQ of CNVs was used as previously described (14). qPCRs were carried out using SYBR green chemistry (SYBRTM Select Master Mix, Applied Biosystems) and the instruments ABI PRISM 7900HT and 7500 real-time PCR system (Applied Biosystems, Inc., Foster City, CA). The reactions were carried out in a 20-μL volume containing 10 ng of genomic DNA. All primers were used at 900 nM. The thermal cycle was 10 min at 95°C, 40 cycles of 15 s at 95°C, and 1 min at 60°C. Each sample was analyzed in quadruplicate. PCR efficiencies (<95%) were evaluated with standard curves, and dissociation curves were drawn for each primer pair to assess for the specificity of the PCRs. Three samples without CNV were used as reference. The results were analyzed with Thermo Fisher Cloud software 1.0 (Applied Biosystems) and qBase Plus v3.2 (Biogazelle). Pearson correlation was employed to evaluate the relationship between the RQ and the estimated nucleotide diversity patterns.

### Identification of microbial signatures.

The identification of ASVs that discriminate samples according to the number of copies of the *ABCC2-DNMBP* loci was performed based on the compositional kernel as implemented the function “classify” of the kernInt R package (71). The classify function runs a supervised classification model based on Support Vector Machine. For that purpose, the available data set was split at random into a training set (80% of data) and a validation set (20%). The *C* hyperparameter’s optimal value was obtained by 10 × 10 cross-validation on the training set. To estimate the mean classification accuracy, the classify function was run 10 times using different training/test splits of the data set. Microbial signatures were obtained from the hyperplane vectorw, and the importance of the ASV was computed by *kernInt*as(wk)^2^ (72). Initially, the top 5% relevant taxa were retained, but a conservative approach was applied afterwards, keeping for subsequent analyses only the ASVs reported as relevant in at least 50% of the replicates. Finally, to identify over-representation at the genus level, the list of selected features was submitted to a taxa-set enrichment analysis (73).

### Data availability.

The raw whole-genome sequencing data have been submitted to the European Nucleotide Archive: ERA19192875. The raw 16S rRNA sequencing data employed in this article has been submitted to the NCBI sequence read archive (https://www.ncbi.nlm.nih.gov/sra) and BioProject: PRJNA608629, and Table S3 includes the corresponding individual biosample accession number under the PRJNA608629 BioProject.
