# Supplementary material for: Copy Number Variation on ABCC2-DNMBP Loci Affects the Diversity and Composition of the Fecal Microbiota in Pigs
Source: Microbiol Spectr. 2023 May 31;11(4):e05271-22. doi: 10.1128/spectrum.05271-22 (PMC10433821; doi:10.1128/spectrum.05271-22)
Supplement: Supplemental file 1 — Supplemental material. Download spectrum.05271-22-s0001.pdf, PDF file, 0.2 MB [file spectrum.05271-22-s0001.pdf]

Table S1. Description of the 531 Copy Number Variant Regions.

CNVR	Chr	start	end	width	freq	type
CNVR1	1	96000	131999	36000	4	loss
CNVR2	1	984000	1019999	36000	8	gain
CNVR3	1	7980000	8015999	36000	1	gain
CNVR4	1	13292000	13331999	40000	9	gain
CNVR5	1	17944000	17995999	52000	8	loss
CNVR6	1	44812000	44847999	36000	2	gain
CNVR7	1	45628000	45663999	36000	2	gain
CNVR8	1	64008000	64047999	40000	1	loss
CNVR9	1	65852000	65887999	36000	3	gain
CNVR10	1	68600000	68639999	40000	4	gain
CNVR11	1	78376000	78415999	40000	3	both
CNVR12	1	81276000	81315999	40000	2	loss
CNVR13	1	81804000	81843999	40000	3	loss
CNVR14	1	82232000	82303999	72000	17	gain
CNVR15	1	84520000	84571999	52000	11	gain
CNVR16	1	92520000	92551999	32000	6	gain
CNVR17	1	104600000	104635999	36000	1	loss
CNVR18	1	126372000	126415999	44000	15	gain
CNVR19	1	129044000	129083999	40000	10	loss
CNVR20	1	136588000	136623999	36000	2	gain
CNVR21	1	136656000	136695999	40000	7	gain
CNVR22	1	142528000	142571999	44000	13	loss
CNVR23	1	153564000	153663999	100000	17	loss
CNVR24	1	157484000	157519999	36000	1	gain
CNVR25	1	167184000	167219999	36000	3	gain
CNVR26	1	176584000	176619999	36000	3	gain
CNVR27	1	177416000	177475999	60000	42	gain
CNVR28	1	187024000	187067999	44000	58	gain
CNVR29	1	189780000	189811999	32000	26	gain
CNVR30	1	194684000	194723999	40000	6	loss
CNVR31	1	196808000	196843999	36000	3	loss
CNVR32	1	199092000	199127999	36000	1	loss
CNVR33	1	199608000	199647999	40000	5	gain
CNVR34	1	200676000	200711999	36000	2	gain
CNVR35	1	203696000	203739999	44000	10	gain
CNVR36	1	203776000	203815999	40000	3	gain
CNVR37	1	220424000	220463999	40000	8	loss
CNVR38	1	241316000	241351999	36000	2	gain
CNVR39	1	242884000	242927999	44000	4	gain
CNVR40	1	247468000	247515999	48000	19	gain

CNVR41	1	253216000	253251999	36000	1	gain
CNVR42	1	253604000	253631999	28000	100	both
CNVR43	1	253604000	253735999	132000	100	both
CNVR44	1	253708000	253735999	28000	100	both
CNVR45	1	253720000	253739999	20000	100	both
CNVR46	1	261972000	261999999	28000	8	gain
CNVR47	1	262660000	262695999	36000	65	both
CNVR48	1	262660000	262835999	176000	100	both
CNVR49	1	262748000	262787999	40000	100	both
CNVR50	1	262748000	262835999	88000	100	both
CNVR51	1	262748000	263019999	272000	100	both
CNVR52	1	262796000	262835999	40000	100	both
CNVR53	1	262820000	262855999	36000	100	both
CNVR54	1	262840000	262887999	48000	23	both
CNVR55	1	262840000	262935999	96000	24	gain
CNVR56	1	262912000	263035999	124000	23	both
CNVR57	1	263052000	263095999	44000	36	both
CNVR58	1	263644000	263703999	60000	7	gain
CNVR59	2	916000	951999	36000	1	gain
CNVR60	2	9340000	9407999	68000	39	both
CNVR61	2	11504000	11539999	36000	1	gain
CNVR62	2	11948000	11975999	28000	2	gain
CNVR63	2	26272000	26307999	36000	3	gain
CNVR64	2	27440000	27483999	44000	3	both
CNVR65	2	28120000	28163999	44000	38	loss
CNVR66	2	33812000	33903999	92000	2	gain
CNVR67	2	36396000	36443999	48000	18	gain
CNVR68	2	38648000	38683999	36000	2	gain
CNVR69	2	40104000	40143999	40000	6	gain
CNVR70	2	48292000	48327999	36000	3	gain
CNVR71	2	52496000	52531999	36000	51	gain
CNVR72	2	53032000	53087999	56000	23	gain
CNVR73	2	53984000	54051999	68000	14	gain
CNVR74	2	54432000	54487999	56000	20	gain
CNVR75	2	57192000	57227999	36000	6	gain
CNVR76	2	57912000	57947999	36000	1	gain
CNVR77	2	83984000	84019999	36000	10	loss
CNVR78	2	84632000	84675999	44000	3	gain
CNVR79	2	89136000	89159999	24000	37	gain
CNVR80	2	89136000	89219999	84000	37	gain
CNVR81	2	94536000	94571999	36000	1	loss
CNVR82	2	102636000	102671999	36000	1	gain
CNVR83	2	104708000	104743999	36000	1	gain

CNVR84	2	110260000	110295999	36000	1	loss
CNVR85	2	111332000	111371999	40000	19	both
CNVR86	2	111400000	111679999	280000	2	loss
CNVR87	2	111880000	111919999	40000	44	loss
CNVR88	2	125076000	125111999	36000	2	loss
CNVR89	2	126104000	126139999	36000	26	loss
CNVR90	2	127752000	129959999	2208000	1	loss
CNVR91	2	131116000	131195999	80000	8	gain
CNVR92	2	133500000	133539999	40000	20	loss
CNVR93	2	141532000	141567999	36000	2	gain
CNVR94	2	142880000	142915999	36000	6	both
CNVR95	2	149088000	149123999	36000	3	gain
CNVR96	2	151140000	151175999	36000	1	gain
CNVR97	2	151808000	151835999	28000	11	both
CNVR98	3	1064000	1099999	36000	2	gain
CNVR99	3	3824000	3867999	44000	22	both
CNVR100	3	6676000	6775999	100000	59	gain
CNVR101	3	6676000	6783999	108000	59	gain
CNVR102	3	6724000	6759999	36000	59	gain
CNVR103	3	9672000	9711999	40000	25	both
CNVR104	3	23392000	23427999	36000	3	gain
CNVR105	3	31068000	31107999	40000	1	gain
CNVR106	3	31272000	31307999	36000	1	gain
CNVR107	3	54976000	55019999	44000	4	gain
CNVR108	3	57092000	57131999	40000	8	loss
CNVR109	3	74404000	74439999	36000	1	gain
CNVR110	3	79416000	79447999	32000	1	gain
CNVR111	3	89328000	89367999	40000	1	loss
CNVR112	3	89792000	89823999	32000	53	gain
CNVR113	3	89792000	89887999	96000	53	gain
CNVR114	3	89804000	89851999	48000	53	gain
CNVR115	3	89836000	89883999	48000	53	gain
CNVR116	3	89860000	89887999	28000	53	gain
CNVR117	3	94112000	94147999	36000	4	loss
CNVR118	3	103708000	103743999	36000	1	gain
CNVR119	3	110360000	110403999	44000	7	gain
CNVR120	3	121880000	121919999	40000	1	gain
CNVR121	3	123124000	123555999	432000	1	gain
CNVR122	3	130188000	130227999	40000	6	gain
CNVR123	4	40000	71999	32000	1	loss
CNVR124	4	840000	879999	40000	6	loss
CNVR125	4	2568000	2603999	36000	3	gain
CNVR126	4	4680000	4715999	36000	1	gain

CNVR127	4	5740000	5815999	76000	42	gain
CNVR128	4	5744000	5767999	24000	42	gain
CNVR129	4	5788000	5811999	24000	42	gain
CNVR130	4	7744000	7783999	40000	28	loss
CNVR131	4	10720000	10751999	32000	3	gain
CNVR132	4	13428000	13463999	36000	2	gain
CNVR133	4	13632000	13667999	36000	2	loss
CNVR134	4	27328000	27367999	40000	8	loss
CNVR135	4	30824000	30863999	40000	3	gain
CNVR136	4	34472000	34515999	44000	25	gain
CNVR137	4	38004000	38031999	28000	1	loss
CNVR138	4	44784000	44827999	44000	12	gain
CNVR139	4	45100000	45139999	40000	29	gain
CNVR140	4	46428000	46455999	28000	55	loss
CNVR141	4	47504000	47539999	36000	40	gain
CNVR142	4	51064000	51099999	36000	1	loss
CNVR143	4	53832000	53871999	40000	24	loss
CNVR144	4	53936000	53971999	36000	57	loss
CNVR145	4	57552000	57595999	44000	6	gain
CNVR146	4	65524000	65571999	48000	5	loss
CNVR147	4	70920000	70955999	36000	4	gain
CNVR148	4	74864000	74903999	40000	3	gain
CNVR149	4	80148000	80319999	172000	9	loss
CNVR150	4	82880000	82915999	36000	1	gain
CNVR151	4	100384000	100451999	68000	14	both
CNVR152	4	104296000	104331999	36000	2	gain
CNVR153	4	110396000	110423999	28000	2	gain
CNVR154	4	116896000	116935999	40000	12	loss
CNVR155	4	126044000	126079999	36000	41	both
CNVR156	4	129416000	129631999	216000	1	loss
CNVR157	5	9264000	9299999	36000	2	gain
CNVR158	5	9652000	9687999	36000	3	gain
CNVR159	5	19900000	19971999	72000	8	both
CNVR160	5	20168000	20203999	36000	10	gain
CNVR161	5	20168000	20567999	400000	74	both
CNVR162	5	20256000	20611999	356000	71	both
CNVR163	5	20368000	20395999	28000	27	gain
CNVR164	5	20368000	20483999	116000	35	gain
CNVR165	5	20460000	20611999	152000	62	both
CNVR166	5	20548000	20611999	64000	61	both
CNVR167	5	20548000	20611999	64000	61	both
CNVR168	5	21052000	21087999	36000	3	gain
CNVR169	5	21980000	22019999	40000	14	gain

CNVR170	5	39776000	40195999	420000	78	both
CNVR171	5	40032000	40091999	60000	9	both
CNVR172	5	40092000	40215999	124000	76	both
CNVR173	5	40176000	40195999	20000	75	both
CNVR174	5	40176000	40215999	40000	75	both
CNVR175	5	44500000	44531999	32000	1	gain
CNVR176	5	63452000	63531999	80000	27	both
CNVR177	5	63456000	63483999	28000	27	both
CNVR178	5	63504000	63531999	28000	27	both
CNVR179	5	80256000	80307999	52000	25	gain
CNVR180	5	87172000	87207999	36000	7	gain
CNVR181	5	87248000	87299999	52000	1	loss
CNVR182	5	90240000	90267999	28000	15	gain
CNVR183	5	91312000	91351999	40000	1	gain
CNVR184	5	94596000	94627999	32000	1	gain
CNVR185	5	95368000	95471999	104000	9	loss
CNVR186	5	100660000	100695999	36000	1	gain
CNVR187	5	101388000	101431999	44000	16	gain
CNVR188	5	102460000	102495999	36000	3	gain
CNVR189	6	12176000	12251999	76000	1	loss
CNVR190	6	14916000	14955999	40000	2	loss
CNVR191	6	15888000	15923999	36000	2	gain
CNVR192	6	18648000	18683999	36000	1	loss
CNVR193	6	21240000	21275999	36000	3	loss
CNVR194	6	29536000	29603999	68000	7	gain
CNVR195	6	34872000	34911999	40000	2	gain
CNVR196	6	39840000	39875999	36000	2	gain
CNVR197	6	44176000	44219999	44000	31	loss
CNVR198	6	46248000	46287999	40000	17	loss
CNVR199	6	57808000	57855999	48000	1	gain
CNVR200	6	59592000	59627999	36000	3	both
CNVR201	6	62096000	62135999	40000	6	gain
CNVR202	6	62144000	62183999	40000	6	loss
CNVR203	6	62460000	62495999	36000	2	gain
CNVR204	6	65540000	65575999	36000	1	gain
CNVR205	6	70012000	70055999	44000	3	gain
CNVR206	6	82324000	82359999	36000	3	gain
CNVR207	6	86980000	87015999	36000	2	gain
CNVR208	6	96164000	96203999	40000	32	both
CNVR209	6	96916000	96951999	36000	3	gain
CNVR210	6	102308000	102343999	36000	3	gain
CNVR211	6	111128000	111163999	36000	1	loss
CNVR212	6	114144000	114179999	36000	2	gain

CNVR213	6	115584000	115619999	36000	2	gain
CNVR214	6	120492000	120527999	36000	3	gain
CNVR215	6	124472000	124511999	40000	5	both
CNVR216	6	124956000	124991999	36000	2	gain
CNVR217	6	125256000	125291999	36000	3	gain
CNVR218	6	126824000	126887999	64000	8	gain
CNVR219	6	142776000	142819999	44000	25	gain
CNVR220	6	145356000	145391999	36000	1	gain
CNVR221	6	151620000	151663999	44000	1	gain
CNVR222	6	156504000	156539999	36000	2	gain
CNVR223	6	158540000	158583999	44000	14	gain
CNVR224	6	163056000	163091999	36000	2	loss
CNVR225	6	163208000	163235999	28000	3	loss
CNVR226	6	165292000	165319999	28000	3	loss
CNVR227	6	165500000	165543999	44000	38	gain
CNVR228	6	166856000	166895999	40000	6	gain
CNVR229	6	168240000	168267999	28000	24	both
CNVR230	7	580000	615999	36000	2	gain
CNVR231	7	4284000	4323999	40000	3	loss
CNVR232	7	6612000	6647999	36000	3	gain
CNVR233	7	10848000	10887999	40000	15	both
CNVR234	7	14944000	14983999	40000	6	both
CNVR235	7	23004000	23043999	40000	11	both
CNVR236	7	26160000	26211999	52000	3	loss
CNVR237	7	31700000	31739999	40000	9	gain
CNVR238	7	45480000	45515999	36000	2	gain
CNVR239	7	47456000	47491999	36000	1	gain
CNVR240	7	48052000	48087999	36000	3	gain
CNVR241	7	58768000	58807999	40000	10	gain
CNVR242	7	58772000	58851999	80000	10	gain
CNVR243	7	58772000	58851999	80000	10	gain
CNVR244	7	58816000	58851999	36000	10	gain
CNVR245	7	60124000	60159999	36000	3	gain
CNVR246	7	69712000	69747999	36000	2	gain
CNVR247	7	73072000	73107999	36000	10	loss
CNVR248	7	74400000	74435999	36000	2	gain
CNVR249	7	76524000	76895999	372000	9	both
CNVR250	7	76548000	76731999	184000	9	both
CNVR251	7	77148000	77183999	36000	1	loss
CNVR252	7	77384000	77427999	44000	64	gain
CNVR253	7	78408000	78447999	40000	5	gain
CNVR254	7	79920000	79951999	32000	4	gain
CNVR255	7	79924000	79987999	64000	4	gain

CNVR256	7	89480000	89519999	40000	6	loss
CNVR257	7	91428000	91463999	36000	9	loss
CNVR258	7	97840000	97911999	72000	24	gain
CNVR259	7	98736000	98795999	60000	24	both
CNVR260	7	98768000	98795999	28000	24	both
CNVR261	7	105604000	105639999	36000	1	gain
CNVR262	7	112404000	112439999	36000	2	gain
CNVR263	7	113060000	113083999	24000	24	loss
CNVR264	7	113060000	113103999	44000	24	loss
CNVR265	7	113084000	113107999	24000	24	loss
CNVR266	8	168000	203999	36000	3	gain
CNVR267	8	4428000	4463999	36000	10	gain
CNVR268	8	5096000	5135999	40000	39	loss
CNVR269	8	13312000	13355999	44000	7	gain
CNVR270	8	25480000	25507999	28000	1	loss
CNVR271	8	34556000	34615999	60000	32	gain
CNVR272	8	41208000	41799999	592000	100	loss
CNVR273	8	42612000	42647999	36000	50	gain
CNVR274	8	53444000	53471999	28000	27	loss
CNVR275	8	53776000	53807999	32000	4	both
CNVR276	8	54656000	54699999	44000	32	gain
CNVR277	8	60484000	60523999	40000	1	loss
CNVR278	8	62440000	62479999	40000	3	loss
CNVR279	8	65820000	65863999	44000	13	gain
CNVR280	8	73120000	73155999	36000	12	both
CNVR281	8	91652000	91691999	40000	2	both
CNVR282	8	93336000	93375999	40000	5	loss
CNVR283	8	98340000	98379999	40000	12	gain
CNVR284	8	98396000	98427999	32000	1	loss
CNVR285	8	99536000	99555999	20000	59	gain
CNVR286	8	99536000	99579999	44000	59	gain
CNVR287	8	99560000	99579999	20000	59	gain
CNVR288	8	104536000	104575999	40000	10	gain
CNVR289	8	105888000	105927999	40000	6	gain
CNVR290	8	107552000	107591999	40000	3	loss
CNVR291	8	117736000	117775999	40000	8	both
CNVR292	8	119576000	119611999	36000	2	gain
CNVR293	8	121900000	121963999	64000	16	gain
CNVR294	8	121904000	121931999	28000	16	gain
CNVR295	8	122016000	122071999	56000	13	gain
CNVR296	8	122920000	122955999	36000	1	gain
CNVR297	8	125096000	125131999	36000	3	gain
CNVR298	8	130668000	130695999	28000	49	both

CNVR299	8	130668000	130727999	60000	49	both
CNVR300	8	130700000	130723999	24000	49	both
CNVR301	8	133432000	133467999	36000	3	gain
CNVR302	8	133988000	134023999	36000	1	loss
CNVR303	8	135592000	135627999	36000	2	gain
CNVR304	9	1816000	1887999	72000	17	both
CNVR305	9	4104000	4147999	44000	5	gain
CNVR306	9	4364000	4403999	40000	5	loss
CNVR307	9	5068000	5115999	48000	1	gain
CNVR308	9	9968000	10003999	36000	88	loss
CNVR309	9	18312000	18359999	48000	14	gain
CNVR310	9	30468000	30503999	36000	1	loss
CNVR311	9	39676000	39711999	36000	7	gain
CNVR312	9	45852000	45887999	36000	3	loss
CNVR313	9	51752000	51779999	28000	6	loss
CNVR314	9	63376000	63403999	28000	2	gain
CNVR315	9	70620000	70651999	32000	93	both
CNVR316	9	70624000	70671999	48000	93	both
CNVR317	9	70628000	70667999	40000	93	both
CNVR318	9	70632000	70651999	20000	93	both
CNVR319	9	70644000	70663999	20000	93	both
CNVR320	9	70644000	70675999	32000	93	both
CNVR321	9	77208000	77239999	32000	1	loss
CNVR322	9	81636000	81687999	52000	2	gain
CNVR323	9	83876000	83915999	40000	8	gain
CNVR324	9	92524000	92619999	96000	1	loss
CNVR325	9	108456000	108491999	36000	2	loss
CNVR326	9	118116000	118143999	28000	45	gain
CNVR327	9	118116000	118227999	112000	45	gain
CNVR328	9	118196000	118223999	28000	45	gain
CNVR329	9	127760000	127779999	20000	3	both
CNVR330	9	136028000	136063999	36000	3	gain
CNVR331	10	2928000	2971999	44000	8	both
CNVR332	10	10348000	10383999	36000	18	loss
CNVR333	10	12656000	12703999	48000	20	gain
CNVR334	10	13432000	13467999	36000	2	gain
CNVR335	10	18828000	18867999	40000	36	both
CNVR336	10	19168000	19195999	28000	21	both
CNVR337	10	19396000	19515999	120000	10	both
CNVR338	10	24916000	24963999	48000	20	gain
CNVR339	10	25820000	25855999	36000	1	gain
CNVR340	10	26288000	26323999	36000	2	loss
CNVR341	10	29700000	29727999	28000	5	both

CNVR342	10	34384000	34419999	36000	1	loss
CNVR343	10	39856000	39891999	36000	5	both
CNVR344	10	40080000	40119999	40000	1	gain
CNVR345	10	50076000	50119999	44000	12	gain
CNVR346	10	58568000	58603999	36000	25	gain
CNVR347	10	58856000	58891999	36000	5	loss
CNVR348	10	59160000	59199999	40000	17	loss
CNVR349	11	488000	523999	36000	2	gain
CNVR350	11	11000000	11035999	36000	1	gain
CNVR351	11	20624000	20663999	40000	4	loss
CNVR352	11	33708000	33743999	36000	1	gain
CNVR353	11	36844000	36879999	36000	1	gain
CNVR354	11	40472000	40511999	40000	3	loss
CNVR355	11	41000000	41027999	28000	1	gain
CNVR356	11	41912000	41955999	44000	5	gain
CNVR357	11	47684000	47719999	36000	5	both
CNVR358	11	56908000	56951999	44000	8	gain
CNVR359	11	56996000	57023999	28000	54	both
CNVR360	11	56996000	57279999	284000	56	both
CNVR361	11	57232000	57267999	36000	56	both
CNVR362	11	57256000	57283999	28000	52	loss
CNVR363	11	66012000	66047999	36000	2	gain
CNVR364	11	73584000	73623999	40000	30	both
CNVR365	12	10684000	10719999	36000	3	gain
CNVR366	12	11488000	11523999	36000	3	gain
CNVR367	12	18060000	18103999	44000	8	loss
CNVR368	12	20848000	20891999	44000	6	gain
CNVR369	12	22360000	22395999	36000	2	gain
CNVR370	12	26564000	26651999	88000	2	gain
CNVR371	12	29472000	29499999	28000	1	gain
CNVR372	12	32268000	32303999	36000	2	gain
CNVR373	12	34280000	34319999	40000	5	gain
CNVR374	12	34360000	34399999	40000	16	gain
CNVR375	12	34872000	34911999	40000	32	both
CNVR376	12	40916000	40951999	36000	3	gain
CNVR377	12	44848000	44883999	36000	3	gain
CNVR378	12	49088000	49119999	32000	49	both
CNVR379	13	5488000	5523999	36000	5	gain
CNVR380	13	6096000	6131999	36000	1	gain
CNVR381	13	6736000	6763999	28000	1	gain
CNVR382	13	13436000	13471999	36000	50	both
CNVR383	13	20596000	20635999	40000	6	both
CNVR384	13	31544000	31583999	40000	100	loss

CNVR385	13	35620000	35663999	44000	16	gain
CNVR386	13	47800000	47843999	44000	2	loss
CNVR387	13	52472000	52507999	36000	5	gain
CNVR388	13	53436000	53479999	44000	8	loss
CNVR389	13	62728000	62751999	24000	28	gain
CNVR390	13	62728000	62783999	56000	28	gain
CNVR391	13	62752000	62775999	24000	28	gain
CNVR392	13	66444000	66475999	32000	37	gain
CNVR393	13	68460000	68495999	36000	4	gain
CNVR394	13	69288000	69327999	40000	3	gain
CNVR395	13	113320000	113359999	40000	7	loss
CNVR396	13	113556000	113579999	24000	25	loss
CNVR397	13	113556000	113603999	48000	25	loss
CNVR398	13	113580000	113607999	28000	25	loss
CNVR399	13	131616000	131679999	64000	20	both
CNVR400	13	141156000	141191999	36000	3	gain
CNVR401	13	148892000	148919999	28000	15	loss
CNVR402	13	148896000	149171999	276000	15	loss
CNVR403	13	149148000	149175999	28000	15	loss
CNVR404	13	160924000	160959999	36000	2	loss
CNVR405	13	169480000	169527999	48000	2	gain
CNVR406	13	172892000	172927999	36000	2	loss
CNVR407	13	175388000	175431999	44000	22	gain
CNVR408	13	184068000	184107999	40000	4	loss
CNVR409	13	196408000	196443999	36000	1	loss
CNVR410	13	196668000	196703999	36000	2	gain
CNVR411	13	198740000	198775999	36000	2	gain
CNVR412	13	203304000	203383999	80000	60	both
CNVR413	13	203308000	203351999	44000	60	both
CNVR414	13	203336000	203371999	36000	60	both
CNVR415	13	205864000	205899999	36000	3	gain
CNVR416	13	205928000	205963999	36000	1	gain
CNVR417	13	206028000	206063999	36000	1	loss
CNVR418	14	1520000	1559999	40000	11	gain
CNVR419	14	1564000	1599999	36000	3	gain
CNVR420	14	1812000	1851999	40000	10	gain
CNVR421	14	4012000	4055999	44000	1	gain
CNVR422	14	7320000	7383999	64000	32	both
CNVR423	14	12744000	12787999	44000	3	gain
CNVR424	14	16496000	16531999	36000	3	gain
CNVR425	14	17452000	17491999	40000	1	loss
CNVR426	14	21340000	21375999	36000	2	gain
CNVR427	14	21792000	21827999	36000	2	loss

CNVR428	14	22000000	22035999	36000	1	gain
CNVR429	14	30512000	30547999	36000	9	both
CNVR430	14	38704000	38739999	36000	2	gain
CNVR431	14	40924000	40951999	28000	1	gain
CNVR432	14	48744000	48799999	56000	38	gain
CNVR433	14	48760000	48799999	40000	38	gain
CNVR434	14	52752000	52839999	88000	9	loss
CNVR435	14	53252000	53287999	36000	1	loss
CNVR436	14	54204000	54243999	40000	17	gain
CNVR437	14	59564000	59599999	36000	2	gain
CNVR438	14	65044000	65079999	36000	2	gain
CNVR439	14	68480000	68523999	44000	1	gain
CNVR440	14	74596000	74631999	36000	2	gain
CNVR441	14	76528000	76563999	36000	1	loss
CNVR442	14	83828000	83863999	36000	13	gain
CNVR443	14	86672000	86707999	36000	3	gain
CNVR444	14	88988000	89055999	68000	26	both
CNVR445	14	92664000	92703999	40000	2	both
CNVR446	14	93752000	93791999	40000	14	gain
CNVR447	14	96956000	97043999	88000	2	both
CNVR448	14	99988000	100023999	36000	2	gain
CNVR449	14	101808000	101851999	44000	8	gain
CNVR450	14	106080000	106195999	116000	22	gain
CNVR451	14	106124000	106371999	248000	70	both
CNVR452	14	106328000	106371999	44000	61	both
CNVR453	14	106328000	106527999	200000	61	both
CNVR454	14	111000000	111075999	76000	52	gain
CNVR455	14	113720000	113755999	36000	51	loss
CNVR456	14	115896000	115931999	36000	2	gain
CNVR457	14	120232000	120267999	36000	2	gain
CNVR458	14	136876000	136923999	48000	3	gain
CNVR459	14	138144000	138195999	52000	9	gain
CNVR460	14	138564000	138599999	36000	2	gain
CNVR461	14	139396000	139431999	36000	1	gain
CNVR462	15	0	135999	136000	27	gain
CNVR463	15	56000	119999	64000	27	gain
CNVR464	15	5992000	6027999	36000	1	gain
CNVR465	15	16012000	16107999	96000	99	loss
CNVR466	15	16072000	16107999	36000	99	loss
CNVR467	15	22464000	22503999	40000	11	gain
CNVR468	15	28964000	28999999	36000	1	gain
CNVR469	15	29972000	30007999	36000	1	gain
CNVR470	15	30280000	30307999	28000	32	gain

CNVR471	15	30280000	30359999	80000	32	gain
CNVR472	15	30336000	30359999	24000	32	gain
CNVR473	15	32056000	32079999	24000	48	both
CNVR474	15	32056000	32103999	48000	48	both
CNVR475	15	32076000	32099999	24000	48	both
CNVR476	15	32500000	32535999	36000	1	gain
CNVR477	15	49688000	49723999	36000	15	gain
CNVR478	15	57012000	57043999	32000	1	gain
CNVR479	15	58064000	58099999	36000	2	gain
CNVR480	15	78200000	78279999	80000	20	both
CNVR481	15	83352000	83387999	36000	2	gain
CNVR482	15	85556000	85591999	36000	3	gain
CNVR483	15	88784000	88839999	56000	9	gain
CNVR484	15	90524000	90587999	64000	40	both
CNVR485	15	90852000	90891999	40000	43	both
CNVR486	15	117136000	117175999	40000	6	gain
CNVR487	15	124704000	124743999	40000	5	gain
CNVR488	15	126740000	126779999	40000	50	loss
CNVR489	15	127196000	127231999	36000	1	gain
CNVR490	15	137720000	137759999	40000	24	gain
CNVR491	16	1208000	1247999	40000	46	both
CNVR492	16	6280000	6319999	40000	21	gain
CNVR493	16	6936000	6971999	36000	4	gain
CNVR494	16	8952000	8991999	40000	10	loss
CNVR495	16	11464000	11511999	48000	21	both
CNVR496	16	18376000	18411999	36000	3	gain
CNVR497	16	36128000	36167999	40000	7	gain
CNVR498	16	39348000	39383999	36000	2	gain
CNVR499	16	47728000	47763999	36000	17	gain
CNVR500	16	50284000	50355999	72000	1	gain
CNVR501	16	59976000	60011999	36000	1	gain
CNVR502	16	61812000	61851999	40000	12	loss
CNVR503	16	65840000	65875999	36000	16	gain
CNVR504	16	68604000	68639999	36000	2	gain
CNVR505	16	73188000	73223999	36000	1	gain
CNVR506	17	0	703999	704000	61	gain
CNVR507	17	372000	423999	52000	60	gain
CNVR508	17	396000	707999	312000	60	gain
CNVR509	17	636000	707999	72000	47	gain
CNVR510	17	676000	707999	32000	47	gain
CNVR511	17	3732000	3775999	44000	31	both
CNVR512	17	4372000	4407999	36000	1	gain
CNVR513	17	11572000	11607999	36000	1	loss

CNVR514	17	11672000	11707999	36000	4	gain
CNVR515	17	13276000	13315999	40000	4	loss
CNVR516	17	13436000	13507999	72000	39	gain
CNVR517	17	34116000	34155999	40000	1	gain
CNVR518	17	53404000	53439999	36000	2	gain
CNVR519	17	62600000	62635999	36000	4	loss
CNVR520	18	252000	291999	40000	11	gain
CNVR521	18	6292000	6327999	36000	2	gain
CNVR522	18	7432000	7563999	132000	1	loss
CNVR523	18	9052000	9087999	36000	2	loss
CNVR524	18	15312000	15347999	36000	1	gain
CNVR525	18	19668000	19703999	36000	1	gain
CNVR526	18	22172000	22215999	44000	9	gain
CNVR527	18	27936000	27975999	40000	3	loss
CNVR528	18	30056000	30095999	40000	2	loss
CNVR529	18	33728000	33767999	40000	2	gain
CNVR530	18	50148000	50183999	36000	1	loss
CNVR531	18	50304000	50339999	36000	1	gain

Figure S1. Correlation coefficients between CNV relative quantification (RQ). the diversity index. and the nucleotide variability of the CNV genomic interval estimators ATajima and RTajima.

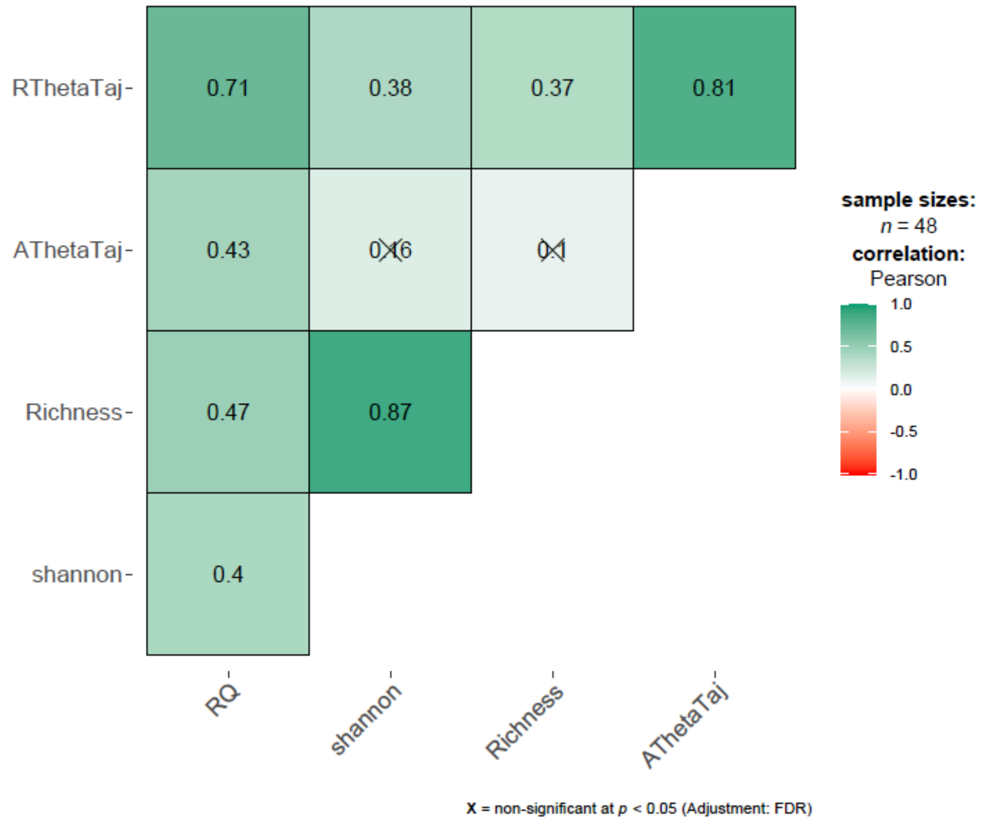

Table S2. Taxonomic composition at family level of the 122 discriminant ASVs.

ASVId	Freq[a]	Phylum	Class	Order	Family
ASV1697	1	Firmicutes	Clostridia	Clostridiales	Ruminococcaceae
ASV1819	1	Firmicutes	Clostridia	Clostridiales	Unassigned
ASV2020	1	Bacteroidetes	Bacteroidia	Bacteroidales	Unassigned
ASV2038	1	Bacteroidetes	Bacteroidia	Bacteroidales	Prevotellaceae
ASV2237	1	Fibrobacteres	Fibrobacteria	Fibrobacterales	Fibrobacteraceae
ASV2273	1	Bacteroidetes	Bacteroidia	Bacteroidales	S24-7
ASV2281	1	Firmicutes	Clostridia	Clostridiales	Ruminococcaceae
ASV2313	1	Bacteroidetes	Bacteroidia	Bacteroidales	S24-7
ASV2433	1	Firmicutes	Clostridia	Clostridiales	Lachnospiraceae
ASV2487	1	Proteobacteria	Gammaproteobacteria	Aeromonadales	Succinivibrionaceae
ASV1449	0.9	Firmicutes	Clostridia	Clostridiales	Lachnospiraceae
ASV1728	0.9	Firmicutes	Clostridia	Clostridiales	Unassigned
ASV1753	0.9	Firmicutes	Clostridia	Clostridiales	Peptococcaceae
ASV1822	0.9	Firmicutes	Clostridia	Clostridiales	Lachnospiraceae
ASV1936	0.9	Firmicutes	Clostridia	Clostridiales	Ruminococcaceae
ASV2081	0.9	Proteobacteria	Deltaproteobacteria	Desulfovibrionales	Desulfovibrionaceae
ASV2124	0.9	Tenericutes	Mollicutes	RF39	Unassigned
ASV2169	0.9	Actinobacteria	Coriobacteriia	Coriobacteriales	Coriobacteriaceae
ASV2171	0.9	Firmicutes	Clostridia	Clostridiales	Lachnospiraceae
ASV2268	0.9	Proteobacteria	Deltaproteobacteria	Desulfovibrionales	Desulfovibrionaceae
ASV2308	0.9	Bacteroidetes	Bacteroidia	Bacteroidales	S24-7
ASV2371	0.9	Firmicutes	Clostridia	Clostridiales	Ruminococcaceae
ASV2378	0.9	Firmicutes	Clostridia	Clostridiales	Ruminococcaceae
ASV2394	0.9	Bacteroidetes	Bacteroidia	Bacteroidales	S24-7
ASV2550	0.9	Bacteroidetes	Bacteroidia	Bacteroidales	S24-7
ASV2567	0.9	Firmicutes	Clostridia	Clostridiales	Ruminococcaceae
ASV1765	0.8	Firmicutes	Clostridia	Clostridiales	Lachnospiraceae
ASV1872	0.8	Firmicutes	Clostridia	Clostridiales	Veillonellaceae
ASV1886	0.8	Firmicutes	Clostridia	Clostridiales	Veillonellaceae
ASV1908	0.8	Bacteroidetes	Bacteroidia	Bacteroidales	Prevotellaceae
ASV1932	0.8	Firmicutes	Clostridia	Clostridiales	Veillonellaceae
ASV1937	0.8	Firmicutes	Clostridia	Clostridiales	Ruminococcaceae
ASV1985	0.8	Firmicutes	Clostridia	Clostridiales	Lachnospiraceae
ASV2028	0.8	Firmicutes	Clostridia	Clostridiales	Veillonellaceae
ASV2144	0.8	Bacteroidetes	Bacteroidia	Bacteroidales	Unassigned
ASV2151	0.8	Firmicutes	Clostridia	Clostridiales	Unassigned
ASV2154	0.8	Firmicutes	Clostridia	Clostridiales	Lachnospiraceae
ASV2325	0.8	Bacteroidetes	Bacteroidia	Bacteroidales	S24-7
ASV2356	0.8	Firmicutes	Clostridia	Clostridiales	Ruminococcaceae

ASV2372	0.8	Bacteroidetes	Bacteroidia	Bacteroidales	Prevotellaceae
ASV2393	0.8	Proteobacteria	Gammaproteobacteria	Aeromonadales	Succinivibrionaceae
ASV2410	0.8	Firmicutes	Bacilli	Lactobacillales	Lactobacillaceae
ASV2452	0.8	Bacteroidetes	Bacteroidia	Bacteroidales	Prevotellaceae
ASV1128	0.7	Firmicutes	Erysipelotrichi	Erysipelotrichales	Erysipelotrichaceae
ASV1169	0.7	Fibrobacteres	Fibrobacteria	Fibrobacterales	Fibrobacteraceae
ASV1305	0.7	Firmicutes	Clostridia	Clostridiales	Unassigned
ASV1370	0.7	Firmicutes	Clostridia	Clostridiales	Unassigned
ASV1644	0.7	Bacteroidetes	Bacteroidia	Bacteroidales	Unassigned
ASV1757	0.7	Firmicutes	Clostridia	Clostridiales	Ruminococcaceae
ASV1850	0.7	Bacteroidetes	Bacteroidia	Bacteroidales	Prevotellaceae
ASV1853	0.7	Firmicutes	Clostridia	Clostridiales	Unassigned
ASV1871	0.7	Proteobacteria	Deltaproteobacteria	Desulfovibrionales	Desulfovibrionaceae
ASV2104	0.7	Bacteroidetes	Bacteroidia	Bacteroidales	Unassigned
ASV2260	0.7	Bacteroidetes	Bacteroidia	Bacteroidales	Prevotellaceae
ASV2361	0.7	Bacteroidetes	Bacteroidia	Bacteroidales	S24-7
ASV2379	0.7	Bacteroidetes	Bacteroidia	Bacteroidales	Prevotellaceae
ASV2406	0.7	Bacteroidetes	Bacteroidia	Bacteroidales	Prevotellaceae
ASV2625	0.7	Bacteroidetes	Bacteroidia	Bacteroidales	S24-7
ASV2641	0.7	Firmicutes	Clostridia	Clostridiales	Ruminococcaceae
ASV2657	0.7	Firmicutes	Erysipelotrichi	Erysipelotrichales	Erysipelotrichaceae
ASV2672	0.7	Bacteroidetes	Bacteroidia	Bacteroidales	Prevotellaceae
ASV681	0.7	Firmicutes	Clostridia	Clostridiales	Ruminococcaceae
ASV693	0.7	Deferribacteres	Deferribacteres	Deferribacterales	Deferribacteraceae
ASV955	0.7	Firmicutes	Clostridia	Clostridiales	Lachnospiraceae
ASV1227	0.6	Firmicutes	Clostridia	Clostridiales	Lachnospiraceae
ASV1683	0.6	Firmicutes	Clostridia	Clostridiales	Ruminococcaceae
ASV1685	0.6	Bacteroidetes	Bacteroidia	Bacteroidales	Prevotellaceae
ASV1759	0.6	Firmicutes	Clostridia	Clostridiales	Ruminococcaceae
ASV1797	0.6	Spirochaetes	Spirochaetes	Spirochaetales	Spirochaetaceae
ASV1953	0.6	Bacteroidetes	Bacteroidia	Bacteroidales	Prevotellaceae
ASV1996	0.6	Firmicutes	Clostridia	Clostridiales	Lachnospiraceae
ASV2064	0.6	Firmicutes	Clostridia	Clostridiales	Unassigned
ASV2189	0.6	Firmicutes	Clostridia	Clostridiales	Veillonellaceae
ASV2231	0.6	Bacteroidetes	Bacteroidia	Bacteroidales	Prevotellaceae
ASV2242	0.6	Firmicutes	Clostridia	Clostridiales	Unassigned
ASV2262	0.6	Firmicutes	Clostridia	Clostridiales	Ruminococcaceae
ASV2278	0.6	Firmicutes	Clostridia	Clostridiales	Lachnospiraceae
ASV2335	0.6	Firmicutes	Clostridia	Clostridiales	Ruminococcaceae
ASV2336	0.6	Firmicutes	Clostridia	Clostridiales	Veillonellaceae
ASV2396	0.6	Firmicutes	Clostridia	Clostridiales	Ruminococcaceae
ASV2397	0.6	Firmicutes	Clostridia	Clostridiales	Unassigned
ASV2408	0.6	Firmicutes	Clostridia	Clostridiales	Ruminococcaceae

ASV2425	0.6	Firmicutes	Clostridia	Clostridiales	Lachnospiraceae
ASV2441	0.6	Firmicutes	Clostridia	Clostridiales	Lachnospiraceae
ASV2453	0.6	Firmicutes	Clostridia	Clostridiales	Lachnospiraceae
ASV2532	0.6	Bacteroidetes	Bacteroidia	Bacteroidales	Prevotellaceae
ASV2673	0.6	Bacteroidetes	Bacteroidia	Bacteroidales	Unassigned
ASV746	0.6	Bacteroidetes	Bacteroidia	Bacteroidales	Prevotellaceae
ASV773	0.6	Firmicutes	Clostridia	Clostridiales	Lachnospiraceae
ASV920	0.6	Firmicutes	Clostridia	Clostridiales	Ruminococcaceae
ASV1258	0.5	Firmicutes	Clostridia	Clostridiales	Ruminococcaceae
ASV1360	0.5	Firmicutes	Clostridia	Clostridiales	Lachnospiraceae
ASV1368	0.5	Firmicutes	Clostridia	Clostridiales	Lachnospiraceae
ASV1404	0.5	Bacteroidetes	Bacteroidia	Bacteroidales	S24-7
ASV1544	0.5	Firmicutes	Clostridia	Clostridiales	Ruminococcaceae
ASV1586	0.5	Bacteroidetes	Bacteroidia	Bacteroidales	Prevotellaceae
ASV1595	0.5	Bacteroidetes	Bacteroidia	Bacteroidales	Unassigned
ASV1642	0.5	Bacteroidetes	Bacteroidia	Bacteroidales	Unassigned
ASV1695	0.5	Firmicutes	Clostridia	Clostridiales	Ruminococcaceae
ASV1846	0.5	Firmicutes	Clostridia	Clostridiales	Lachnospiraceae
ASV1906	0.5	Bacteroidetes	Bacteroidia	Bacteroidales	Unassigned
ASV1958	0.5	Bacteroidetes	Bacteroidia	Bacteroidales	Prevotellaceae
ASV1966	0.5	Firmicutes	Clostridia	Clostridiales	Veillonellaceae
ASV2000	0.5	Firmicutes	Clostridia	Clostridiales	Veillonellaceae
ASV2086	0.5	Bacteroidetes	Bacteroidia	Bacteroidales	Prevotellaceae
ASV2116	0.5	Bacteroidetes	Bacteroidia	Bacteroidales	Unassigned
ASV2147	0.5	Firmicutes	Clostridia	Clostridiales	Veillonellaceae
ASV2188	0.5	Firmicutes	Clostridia	Clostridiales	Ruminococcaceae
ASV2227	0.5	Bacteroidetes	Bacteroidia	Bacteroidales	Prevotellaceae
ASV2241	0.5	Bacteroidetes	Bacteroidia	Bacteroidales	Unassigned
ASV2255	0.5	Firmicutes	Clostridia	Clostridiales	Lachnospiraceae
ASV2275	0.5	Firmicutes	Clostridia	Clostridiales	Lachnospiraceae
ASV2284	0.5	Firmicutes	Clostridia	Clostridiales	Unassigned
ASV2320	0.5	Bacteroidetes	Bacteroidia	Bacteroidales	Prevotellaceae
ASV2388	0.5	Firmicutes	Bacilli	Lactobacillales	Streptococcaceae
ASV2412	0.5	Firmicutes	Clostridia	Clostridiales	Ruminococcaceae
ASV2424	0.5	Bacteroidetes	Bacteroidia	Bacteroidales	Unassigned
ASV2591	0.5	Firmicutes	Clostridia	Clostridiales	Lachnospiraceae
ASV2610	0.5	Firmicutes	Clostridia	Clostridiales	Veillonellaceae
ASV2683	0.5	Firmicutes	Clostridia	Clostridiales	Lachnospiraceae
ASV646	0.5	Firmicutes	Erysipelotrichi	Erysipelotrichales	Erysipelotrichaceae
ASV770	0.5	Bacteroidetes	Bacteroidia	Bacteroidales	Prevotellaceae

Freq: Indicate the frequency of ASVs selection across replicates in the supervised classification model based on Support Vector Machine.

Table S3. Biosample accession number of the 100 employed samples within the BioProject: PRJNA608629.

**Biosample_accession**	**library_strategy**	**library_layout**	**instrument_model**	**filetype**
SAMN16967268	AMPLICON	paired	Illumina NovaSeq 6000	fastq
SAMN16967276	AMPLICON	paired	Illumina NovaSeq 6000	fastq
SAMN16967280	AMPLICON	paired	Illumina NovaSeq 6000	fastq
SAMN16967282	AMPLICON	paired	Illumina NovaSeq 6000	fastq
SAMN16967283	AMPLICON	paired	Illumina NovaSeq 6000	fastq
SAMN16967284	AMPLICON	paired	Illumina NovaSeq 6000	fastq
SAMN16967286	AMPLICON	paired	Illumina NovaSeq 6000	fastq
SAMN16967287	AMPLICON	paired	Illumina NovaSeq 6000	fastq
SAMN16967288	AMPLICON	paired	Illumina NovaSeq 6000	fastq
SAMN16967289	AMPLICON	paired	Illumina NovaSeq 6000	fastq
SAMN16967292	AMPLICON	paired	Illumina NovaSeq 6000	fastq
SAMN16967293	AMPLICON	paired	Illumina NovaSeq 6000	fastq
SAMN16967294	AMPLICON	paired	Illumina NovaSeq 6000	fastq
SAMN16967297	AMPLICON	paired	Illumina NovaSeq 6000	fastq
SAMN16967300	AMPLICON	paired	Illumina NovaSeq 6000	fastq
SAMN16967303	AMPLICON	paired	Illumina NovaSeq 6000	fastq
SAMN16967304	AMPLICON	paired	Illumina NovaSeq 6000	fastq
SAMN16967305	AMPLICON	paired	Illumina NovaSeq 6000	fastq
SAMN16967306	AMPLICON	paired	Illumina NovaSeq 6000	fastq
SAMN16967307	AMPLICON	paired	Illumina NovaSeq 6000	fastq
SAMN16967308	AMPLICON	paired	Illumina NovaSeq 6000	fastq
SAMN16967309	AMPLICON	paired	Illumina NovaSeq 6000	fastq
SAMN16967313	AMPLICON	paired	Illumina NovaSeq 6000	fastq
SAMN16967315	AMPLICON	paired	Illumina NovaSeq 6000	fastq
SAMN16967316	AMPLICON	paired	Illumina NovaSeq 6000	fastq
SAMN16967322	AMPLICON	paired	Illumina NovaSeq 6000	fastq
SAMN16967324	AMPLICON	paired	Illumina NovaSeq 6000	fastq
SAMN16967325	AMPLICON	paired	Illumina NovaSeq 6000	fastq
SAMN16967326	AMPLICON	paired	Illumina NovaSeq 6000	fastq
SAMN16967327	AMPLICON	paired	Illumina NovaSeq 6000	fastq
SAMN16967328	AMPLICON	paired	Illumina NovaSeq 6000	fastq
SAMN16967331	AMPLICON	paired	Illumina NovaSeq 6000	fastq
SAMN16967333	AMPLICON	paired	Illumina NovaSeq 6000	fastq
SAMN16967334	AMPLICON	paired	Illumina NovaSeq 6000	fastq
SAMN16967340	AMPLICON	paired	Illumina NovaSeq 6000	fastq
SAMN16967341	AMPLICON	paired	Illumina NovaSeq 6000	fastq
SAMN16967343	AMPLICON	paired	Illumina NovaSeq 6000	fastq
SAMN16967346	AMPLICON	paired	Illumina NovaSeq 6000	fastq
SAMN16967347	AMPLICON	paired	Illumina NovaSeq 6000	fastq
SAMN16967348	AMPLICON	paired	Illumina NovaSeq 6000	fastq
SAMN16967349	AMPLICON	paired	Illumina NovaSeq 6000	fastq
SAMN16967350	AMPLICON	paired	Illumina NovaSeq 6000	fastq
SAMN16967352	AMPLICON	paired	Illumina NovaSeq 6000	fastq

SAMN16967358	AMPLICON	paired	Illumina NovaSeq 6000	fastq
SAMN16967360	AMPLICON	paired	Illumina NovaSeq 6000	fastq
SAMN16967363	AMPLICON	paired	Illumina NovaSeq 6000	fastq
SAMN16967365	AMPLICON	paired	Illumina NovaSeq 6000	fastq
SAMN16967368	AMPLICON	paired	Illumina NovaSeq 6000	fastq
SAMN16967370	AMPLICON	paired	Illumina NovaSeq 6000	fastq
SAMN16967371	AMPLICON	paired	Illumina NovaSeq 6000	fastq
SAMN16967373	AMPLICON	paired	Illumina NovaSeq 6000	fastq
SAMN16967377	AMPLICON	paired	Illumina NovaSeq 6000	fastq
SAMN16967378	AMPLICON	paired	Illumina NovaSeq 6000	fastq
SAMN16967379	AMPLICON	paired	Illumina NovaSeq 6000	fastq
SAMN16967381	AMPLICON	paired	Illumina NovaSeq 6000	fastq
SAMN16967382	AMPLICON	paired	Illumina NovaSeq 6000	fastq
SAMN16967384	AMPLICON	paired	Illumina NovaSeq 6000	fastq
SAMN16967385	AMPLICON	paired	Illumina NovaSeq 6000	fastq
SAMN16967391	AMPLICON	paired	Illumina NovaSeq 6000	fastq
SAMN16967392	AMPLICON	paired	Illumina NovaSeq 6000	fastq
SAMN16967395	AMPLICON	paired	Illumina NovaSeq 6000	fastq
SAMN16967399	AMPLICON	paired	Illumina NovaSeq 6000	fastq
SAMN16967401	AMPLICON	paired	Illumina NovaSeq 6000	fastq
SAMN16967404	AMPLICON	paired	Illumina NovaSeq 6000	fastq
SAMN16967405	AMPLICON	paired	Illumina NovaSeq 6000	fastq
SAMN16967407	AMPLICON	paired	Illumina NovaSeq 6000	fastq
SAMN16967409	AMPLICON	paired	Illumina NovaSeq 6000	fastq
SAMN16967412	AMPLICON	paired	Illumina NovaSeq 6000	fastq
SAMN16967413	AMPLICON	paired	Illumina NovaSeq 6000	fastq
SAMN16967414	AMPLICON	paired	Illumina NovaSeq 6000	fastq
SAMN16967415	AMPLICON	paired	Illumina NovaSeq 6000	fastq
SAMN16967418	AMPLICON	paired	Illumina NovaSeq 6000	fastq
SAMN16967428	AMPLICON	paired	Illumina NovaSeq 6000	fastq
SAMN16967429	AMPLICON	paired	Illumina NovaSeq 6000	fastq
SAMN16967430	AMPLICON	paired	Illumina NovaSeq 6000	fastq
SAMN16967432	AMPLICON	paired	Illumina NovaSeq 6000	fastq
SAMN16967433	AMPLICON	paired	Illumina NovaSeq 6000	fastq
SAMN16967435	AMPLICON	paired	Illumina NovaSeq 6000	fastq
SAMN16967436	AMPLICON	paired	Illumina NovaSeq 6000	fastq
SAMN16967438	AMPLICON	paired	Illumina NovaSeq 6000	fastq
SAMN16967440	AMPLICON	paired	Illumina NovaSeq 6000	fastq
SAMN16967441	AMPLICON	paired	Illumina NovaSeq 6000	fastq
SAMN16967444	AMPLICON	paired	Illumina NovaSeq 6000	fastq
SAMN16967445	AMPLICON	paired	Illumina NovaSeq 6000	fastq
SAMN16967447	AMPLICON	paired	Illumina NovaSeq 6000	fastq
SAMN16967449	AMPLICON	paired	Illumina NovaSeq 6000	fastq
SAMN16967452	AMPLICON	paired	Illumina NovaSeq 6000	fastq
SAMN16967456	AMPLICON	paired	Illumina NovaSeq 6000	fastq
SAMN16967462	AMPLICON	paired	Illumina NovaSeq 6000	fastq
SAMN16967464	AMPLICON	paired	Illumina NovaSeq 6000	fastq

SAMN16967465	AMPLICON	paired	Illumina NovaSeq 6000	fastq
SAMN16967468	AMPLICON	paired	Illumina NovaSeq 6000	fastq
SAMN16967472	AMPLICON	paired	Illumina NovaSeq 6000	fastq
SAMN16967476	AMPLICON	paired	Illumina NovaSeq 6000	fastq
SAMN16967477	AMPLICON	paired	Illumina NovaSeq 6000	fastq
SAMN16967478	AMPLICON	paired	Illumina NovaSeq 6000	fastq
SAMN16967479	AMPLICON	paired	Illumina NovaSeq 6000	fastq
SAMN16967480	AMPLICON	paired	Illumina NovaSeq 6000	fastq
SAMN16967481	AMPLICON	paired	Illumina NovaSeq 6000	fastq
SAMN16967482	AMPLICON	paired	Illumina NovaSeq 6000	fastq
